# Interim report on the effective intraperitoneal therapy of insulin-dependent diabetes mellitus in pet dogs using “Neo-Islets,” aggregates of adipose stem and pancreatic islet cells (INAD 012-776)

**DOI:** 10.1371/journal.pone.0218688

**Published:** 2019-09-19

**Authors:** Anna Gooch, Ping Zhang, Zhuma Hu, Natasha Loy Son, Nicole Avila, Julie Fischer, Gregory Roberts, Rance Sellon, Christof Westenfelder

**Affiliations:** 1 SymbioCellTech, LLC, Salt Lake City, Utah, United States of America; 2 Veterinary Specialty Hospital, San Diego, California, United States of America; 3 Department of Veterinary Clinical Sciences, Washington State University, Pullman, Washington, United States of America; 4 Department of Medicine, Division of Nephrology, University of Utah, Salt Lake City, Utah, United States of America; Centre National de la Recherche Scientifique, FRANCE

## Abstract

We previously reported that allogeneic, intraperitoneally administered “Neo-Islets,” composed of cultured pancreatic islet cells co-aggregated with high numbers of immunoprotective and cytoprotective Adipose-derived Stem Cells, reestablished, through omental engraftment, redifferentiation and splenic and omental up-regulation of regulatory T-cells, normoglycemia in autoimmune Type-1 Diabetic Non-Obese Diabetic (NOD) mice without the use of immunosuppressive agents or encapsulation devices. Based on these observations, we are currently testing this Neo-Islet technology in an FDA guided pilot study (INAD 012–776) in insulin-dependent, spontaneously diabetic pet dogs by ultrasound-guided, intraperitoneal administration of 2x10e5 Neo-Islets/kilogram body weight to metabolically controlled (blood glucose, triglycerides, thyroid and adrenal functions) and sedated animals. We report here interim observations on the first 4 canine Neo-Islet-treated, insulin-dependent pet dogs that are now in the early to intermediate-term follow-up phase of the planned 3 year study (> 6 months post treatment). Current results from this translational study indicate that in dogs, Neo-Islets appear to engraft, redifferentiate and physiologically produce insulin, and are rejected by neither auto- nor allo-immune responses, as evidenced by (a) an absent IgG response to the allogeneic cells contained in the administered Neo-Islets, and (b) progressively improved glycemic control that achieves up to a 50% reduction in daily insulin needs paralleled by a statistically significant decrease in serum glucose concentrations. This is accomplished without the use of anti-rejection drugs or encapsulation devices. No adverse or serious adverse events related to the Neo-Islet administration have been observed to date. We conclude that this minimally invasive therapy has significant translational relevance to veterinary and clinical Type 1 diabetes mellitus by achieving complete and at this point partial glycemic control in two species, i.e., diabetic mice and dogs, respectively.

## Introduction

Diabetes mellitus (DM) is a common endocrine disorder in dogs, and it is estimated that there are currently 700,000 insulin-dependent pet dogs with DM in the US [1–4]. Their care is burdensome and expensive for their owners. As in humans, Type 1 (T1DM) in dogs is generally irreversible, and is caused by lack of insulin secretion in response to glucose, resulting in hyperglycemia, acid-base and electrolyte disorders, polydipsia, polyuria and weight loss, and is accompanied by a broad spectrum of diabetes-induced end organ damage and other complications, including blindness due to retinopathy and cataracts, opportunistic infections, neurological and other serious micro- and macro-vascular complications [4–6]. Although dogs were the model in which insulin was originally discovered, and remain a major large animal model for the refinement of diabetic treatments such as pancreas and islet cell transplants, almost no advances in the treatment of diabetic dogs have been made in the last 50 years [7]. A few studies have examined xeno- or allogeneic islet transplantation to reverse or ameliorate DM in dogs and have had varying degrees of success. Yet, insulin replacement therapy and blood glucose monitoring remain the only currently available therapy for these animals [7–9]. Due to the challenges of medically managing a diabetic dog, up to 40% of owners opt to euthanize their dogs within a day of diagnosis rather than treat them [1,10].

While the pathogenic mechanisms of canine T1DM are still incompletely understood, there is evidence that autoimmune injury of pancreatic beta cells plays a role in approximately 1/3 of cases [3,4,7,10–12]. T1DM occurs with equal frequency in male and female neutered dogs, but as with Non-Obese Diabetic (NOD) mice, at higher frequency in intact females vs. males, suggesting a role for female hormones in the development of the disease in dogs [1,3,13–15]. While T1DM affects both juvenile and adult dogs [1,3,7], it is more commonly seen in adults, generally diagnosed between the ages of 3 and 15 years [3,10]. Some groups have reported isolation of auto-antibodies to proinsulin, GAD65 and IA-2 from the sera of diabetic animals [16,17]. Others, however, have been unable to confirm the presence of such auto-antibodies in the same, previously tested sera, or in sera from other diabetic dogs [2,14]. On the other hand, several studies have found a genetic association between certain dog leukocyte antigen alleles (DLA) and the development of DM in dogs, similar to that found between HLA alleles and the development of DM in humans [12,18,19]. Despite some controversy as to the contribution of immune-mediated destruction of beta cells to canine DM, all pioneering work on islet and pancreas transplantation for humans was carried out in dogs and clearly demonstrated the need for immunosuppression or immune-isolation, as well as sufficient nutrition/oxygenation and vascularization for islet allo-graft survival [7,20].

Dog survival time post the diagnosis of diabetes is short [1,10]. In one study, median post-diagnosis survival time was only 57 days, due either to pet owners’ unwillingness to care for a diabetic animal, or to the dog suffering from advanced stages of diabetic complications at the time of diagnosis. For dogs surviving beyond the first day after diagnosis, the median survival time was 2 years [1]. These low survival rates, and clear unwillingness of some owners to care for diabetic animals, underscore the need for novel and effective therapeutics that remove much of the burden of diabetes treatment and maintenance from pet owners, and to facilitate the survival of affected dogs.

We previously demonstrated that allogeneic, intraperitoneally (i.p.) administered “Neo-Islets” (NIs), composed of culture expanded islet cells (ICs) that have undergone partial Epithelial-Mesenchymal transition, co-aggregated with high numbers of immunoprotective and cytoprotective Adipose-derived Stem Cells (ASCs), could reestablish normoglycemia in NOD mice with autoimmune T1DM without the use of encapsulation devices or immunosuppressive agents [21]. Glycemic control was similarly achieved using dog-derived ASCs and ICs in a Streptozotocin (STZ) model of diabetes in NOD/SCID mice [21]. Dose finding studies in this model indicated that 2x10e5 canine Neo-Islets (cNIs) per kg body weight (b.wt.) given i.p. would be sufficient to control blood glucose levels [21].

Based on these studies, we initiated an FDA-CVM guided pilot study (INAD 012–776) to assess the (i) safety, (ii) feasibility and (iii) efficacy of allogeneic cNIs in significantly reducing or eliminating the need for exogenous insulin in spontaneously diabetic, insulin-dependent pet dogs. We further assessed whether the administered cNIs elicited an allo-immune response. This pilot study is currently ongoing at Veterinary Specialty Hospital in San Diego, CA, and at the Veterinary Teaching Hospital, Washington State University in Pullman, WA. In this translational study, six dogs have been treated and four followed for more than 6 months. We report here on the course of the first four cNI treated dogs that have been followed for more than 6 months. The overall rationale of demonstrating that this cNI therapy is also effective in a second, larger diabetic mammal, the dog, is the fact that this will further strengthen the justification for the currently planned conduct of a clinical trial in human study subjects with T1DM.

## Materials and methods

### Reagents

Reagents used and their manufacturers are listed as indicated below, except for PCR reagents and primers which are listed in S1 Table.

### Study design

An FDA guided pilot study (INAD 012–776) was developed with IACUC approval at (a) Washington State University in Pullman, WA (WSU) and (b) the Veterinary Specialty Hospital in San Diego, CA (VSH). The overall study goal is to include ten insulin dependent pet dogs and follow them for three years post-cNI infusion. Seven dogs have been enrolled according to the criteria in Table 1. Informed consent was obtained from all dog owners prior to enrollment. One owner withdrew her dog prior to treatment, 6 dogs have been treated, and 4 of those (VSH-01, VSH-02, WSU-01 and WSU-02) have been followed for 6 months or longer. Enrolled dogs’ demographics are shown in Table 2 and comorbidities in Table 3. The four dogs discussed in this manuscript were screened and are being followed as shown in S2 Table. It should be noted that although no treated dog is currently 3 years post cNI infusion, this table summarizes the entire follow-up plan as approved for this pilot study by the FDA and IACUC. Pre-treatment serum samples from treated dogs were tested for the presence of islet autoantibodies (see below for details), and all dogs were examined for comorbidities. Prior to treatment, blood glucose, triglycerides and other important metabolic factors (thyroid, pancreas, and adrenal function) were assessed and treated as necessary. After blood glucose and triglyceride levels were adequately controlled (stable blood glucose levels ≤ 350; fasting triglycerides between 20–300 mg/dL), 2x10e5 allogeneic cNIs per kg b.wt. were given i.p.. In all animals, blood glucose and fructosamine concentrations, insulin requirements, body weights, food and fluid intake, formation of antibodies to allogeneic cNIs, animal activity and the development of adverse events were closely monitored by the owners, PIs and the primary veterinarians for each dog.

**Table 1:** Enrollment criteria.

Enrollment Criteria	Insulin dependent, diabetic dog on established insulin and diet regimen Weight between 5 and 12 kg Spayed if female Ability of the dogs’ owners to follow the study protocol, requiring close monitoring of blood glucose concentrations, recording of insulin dosing, body weights and other daily activities.
Exclusion Criteria	History of malignancy Significant illness unrelated to the diabetic state such that the PI believes the dog to be a poor candidate Significantly advanced age such that the PI believes the dog to be a poor candidate Contraindication to utilized sedatives and other drugs Participation in another, ongoing clinical trial
These specific diabetes-associated complications are documented, but do not constitute Exclusion Criteria	Presence of: End-organ damage Cataracts Neuropathy Renal disease History of pancreatitis

**Table 2:** Demographics of enrolled study subjects.

Dog #	Treated	Sex	Breed	Weight* (kg)	Age* (yrs)	Yrs of DM**	Insulin dose**
VSH-01	yes	M	French Bulldog	12.3	9	2.5	4.5–5.5 U Novolin BID
VSH-02	yes	M	Bichon Mix	6.9	7	0.5	5 U Vetsulin BID
VSH-03	yes	F	Bichon Poodle Mix	5.8	11	2	7.5 U Vetsulin BID
VSH-04	yes	M	Chihuahua Mix	7.3	12	1	9 U Vetsulin BID
VSH-05	withdrawn	M	Chihuahua Mix	7	7	1	6 U Vetsulin BID
WSU-01	yes	F	American Eskimo	11	1.8	0.8	2.5 U Vetsulin BID
WSU-02	yes	M	Chihuahua Mix	7	1	0.5	4 U Vetsulin BID

Abbreviations: U = Units, WSU = Washington State University, VSH = Veterinary Specialty Hospital, BID = twice per day. Insulin is listed as per dose. The total daily dose is double that given in the table.

*, at enrollment

**, at time of treatment

**Table 3:** Clinical pretreatment data on dogs that are ≥ 6 months post-cNI treatment.

**VSH-01** • Male, neutered (DOB 8/8/08) • No history of pancreatitis, but evidence on screening abdominal ultrasound (US) of suspected chronic or previous pancreatitis • Cataracts; moderate adrenomegaly, mild renal degenerative changes • HbA1c pretreatment, 6.9% • Fructosamine pretreatment, 366 μmol/L	**VSH-02**Male, neutered (DOB 3/28/11) • No history of pancreatitis, but evidence on screening abdominal US of suspected chronic or previous pancreatitis • Hypothyroid (on 0.1 mg Synthroid BID), diagnosed through screening; mild renal degenerative changes • HbA1c pretreatment, 12.1% • Fructosamine pretreatment, 439 μmol/L
**WSU-01**Female, spayed (DOB 6/15/16) • No history nor evidence of pancreatitis • Hypercholesterolemia • History of liver disease • HbA1c pretreatment, 7.7% • Fructosamine pretreatment, 301 μmol/L	**WSU-02**Male, neutered (DOB 4/15/17) • No history nor evidence of pancreatitis • HbA1c pretreatment, 8.4 • Fructosamine pretreatment, 281 μmol/L

### Blood glucose levels

After infusion of cNIs, blood glucose concentrations were assessed by the owner at least twice daily (morning and evening) using an *AlphaTrak* glucometer or continuously monitored using a *FreeStyle Libre* glucometer in order to guide insulin administration. Insulin (Vetsulin or Novolin) was administered s.c. BID based on blood glucose readings, and the doses given were recorded by the owner and reported to the PIs.

### Cells

#### Cell donor information

All cells, ASCs and ICs, for the study were obtained through an NIH sharing agreement from 27 dogs being euthanized under a University of Utah IACUC approved protocol. This study involved no use of test agents, but did involve the use of a surgically implanted pacemaker in some dogs to induce heart failure, as well as the use of resynchronization therapy via the pacemaker as an experimental treatment. Dogs were up-to-date on vaccinations at the time of euthanasia.

#### Islets and ASCs

Islets and ASCs were isolated and cultured from dogs as previously reported [21–26], and as described in detail in the Supporting Information section of our previous publication [21]. Prior to cNI formation, cultured ASCs were characterized for their ability to undergo trilineage (adipo-, osteo-, chondrogenic) differentiation as described [21] and for surface marker expression of CD90, CD44, CD34, CD45 and DLA-DR as in our previous publication [27], and using the following antibodies: phycoerythrin (PE)-labeled, monoclonal, rat anti-dog CD90 IgG2b, and isotype control (Invitrogen, 12–5900 and 12-4031-83); allophycocyanin (APC)-labeled, monoclonal, mouse anti-dog CD44 IgG1, and isotype control (R&D, FAB5449A and IC002A); PE-labeled, monoclonal, mouse anti-dog CD34 IgG1, and isotype control (BD Biosciences, 559369 and 554680); R-PE-labeled, monoclonal, rat anti-dog CD45 IgG2b, and isotype control (BioRad MCA1042PE and PA5-33195), PE-labeled, monoclonal, mouse anti-human HLA-DR IgG2a (cross-reacts with dog), and isotype control (BD Biosciences 555812 and 555574). All antibodies were used at the concentrations recommended by their respective manufacturers. Prior to use, culture expanded cell lines (ASCs and ICs) were karyotyped by an independent laboratory and found to be normal (Laboratory of Molecular Cytogenics and Genomics, Veterinary Medicine and Biomedical Sciences, Texas A & M University, College Station, Texas).

#### Cell banking

Passage 0 (P0) cultured islet cells and P2 ASCs were suspended in CryoStor CS10 (BioLife Solutions, 210102) and banked, frozen in liquid nitrogen (-140°C) until ready for final expansion and cNI formation. Prior to freezing, cells were release tested for viability, sterility, endotoxin, *Mycoplasma*, expression of various genes involved in immune modulation, cell survival and angiogenesis (see S1 Table), and dog-specific adventitious agents (see Table 4).

**Table 4:** Release criteria for cells and Neo-Islets.

	Viability	Sterility	Endotoxin	*Mycoplasma*	Adventitious Agents	Gene Expression (rtPCR)	Extracellular Marker (FACS; % positive)
**ASCs**	≥ 70%	No growth after 14 days	< 5.0 EU/ml	B/A <1.2	negative for all tested	Significant increase in Ido 1 expression upon overnight culture with 10 ng/ml INF-γ	≥ 90% CD90 and CD44 ≤ 4% CD34, CD45, and DLA-DR
**Islet Cells**	≥ 70%	No growth after 14 days	< 5.0 EU/ml	B/A <1.2	negative for all tested	+ Ins	NA
**Neo-Islets**	≥ 70%	negative Gram stain; no growth after 14 days	< 5.0 EU/ml	B/A <1.2	NA	+ Ins	NA

Adventitious agents tested:

*Neorickettsia risticii*, *Mycoplasma haemocanis*, *Mycoplasma canis*, *Bartonella henselae*, *B*. *bacilliformis*, *B*. *clarridgeiae*, *B*. *elizabethae*, *B*. *quintana and B*. *vinsonii subsp*. *Berkhoffii*, *Brucella abortus*, *B*. *microti*, *B*. *melitensis*, *B*. *pinnipedialis*, *B*. *suis*, *B*. *canis*, *B*. *ovis and B*. *neotomae*, *Neorickettsia helmintheca*, *Cryptococcus neoformans*, Influenza A H5N1, H5N2, H1N1, H2N2, H3N8, H4N6, H7N7, H8N4 and H9N2, canine herpesvirus, *Toxoplasma gondii*

### Real time PCR (rtPCR)

rtPCR was carried out as described in our previous publication [21] using the reagents and primers listed in S1 Table. In brief, Relative Quantification, (RQ; defined as is standard as 2^-ΔΔCT^ where CT is the Cycle Threshold [28]), was calculated through normalization to internal (deltaCT; beta actin and beta 2 microglobulin) and external controls (delta-deltaCT; parent cells), both accomplished using the ABS 7500 Real Time PCR System and software. Results are presented as log10(RQ) ± log10(RQmin and RQmax) so that up- and down- gene regulation is represented equally [29]. Differences between expression levels greater than log10(RQ) 2 or log10(RQ) -2 were considered significant [29].

### Final product (cNI) formation and storage

cNIs were formed in ultra-low adherent 10-layer Cell Stacks (Corning, custom made product) from freshly cultured banked ICs and ASCs using 70x10e6 cells per layer and 140 ml of DMEM (Gibco 11885–084) + 10% dog serum (Golden West) as described in our previous publication [21]. cNIs were harvested and resuspended in 50 to 100 ml of sterile Plasmalyte A (Baxter, 2B2543) + 2% HEPES (Gibco), pH 7.4 at a concentration of 2x10e7 clustered cells/ml, and placed in a sterile 100 ml syringe (Wilburn Medical, WUSA/100). The final product was release tested (viability, sterility, endotoxin, *Mycoplasma*, gram stain, gene expression of insulin (INS), glucagon (GCG), somatostatin (SST), pancreatic polypeptide (PPY), pancreatic and duodenal homeobox 1 (PDX1), vascular endothelial growth factor A (VEGFa), stromal cell derived factor 1 (CXCL12), and stored and transported to the study site at 4°C for administration within 48 hrs of packaging.

### Cell bank and final product release testing

Cell viability was assessed using fluorescein diacetate (Sigma, F7378) and propidium iodide (Life Technologies P3566) as per the manufacturers’ instructions. Sterility was assessed as described in 21 *CFR 610*.*12*, using Tryptic Soy Broth (Sigma, 22092) and Fluid Thioglycollate Medium (Sigma, T9032) and following the manufacturer’s instructions. Endotoxin levels were determined using the Charles River Endosafe Nexgen PTS system and reagents (Charles River) following the manufacturer’s instructions. Possible *Mycoplasma* contamination was assessed using the MycoAlert PLUS *Mycoplasma* Detection Kit (Lonza, LT07-701 and LT07-518) per the manufacturer’s instructions. Samples of banked cells from each donor dog were sent to Zoologix for relevant adventitious agent testing. Prior to cNI formation, ASCs were assessed for expression of genes involved in immune modulation, cell survival and angiogenesis (see S1 Table). ICs were analyzed for expression of islet hormone associated genes. Gram staining was conducted by standard methods using a kit (Sigma, 77730-1KT-F). Cell and cNI release criteria are listed in Table 4.

### Testing of treated dogs’ sera for antibodies to Islet Cells and ASCs

Prior to and at ~6 weeks post cNI treatment, 1 ml of serum was collected from each study dog. 5x10e4 banked allogeneic ASCs, and (separately) 5x10e4 banked allogeneic ICs were incubated for 30 min at room temperature with 100 uL serum each. Cells were then centrifuged at 600 x g for 5 min; the supernatant was removed and the cell pellets resuspended in 200 uL Phosphate Buffered Saline (PBS, Sigma, 11666789001) +1% Fetal Bovine Serum (FBS, GEHealthcare, SH30910.03). Cells were then incubated at room temperature for 30 min with a 1:200 dilution of FITC-labeled, polyclonal rabbit anti-dog IgG or isotype control (Jackson ImmunoResearch; 304-095-003, lot #13971 and 011-090-003, lot #127025 respectively). They were then centrifuged, washed once with PBS + 1% FBS; resuspended in 400 uL PBS + 1% FBS, and analyzed by FACS as previously described [21].

### Enzyme linked immunosorbent assays (ELISAs)

ELISAs for GAD65 and IA2 autoantibodies were kindly carried out by Dr. Boris Fehse, University of Hamburg, Germany, using GAD65 and IA2 ELISA kits (both from Euroimmun, Lübeck, Germany, EA 1022–9601 G and EA 1023–9601 G, respectively) and the same serum samples were analyzed for allo-antibody levels. The GAD65 kit has previously been shown to cross-react with dog antibodies [30].

### cNI Administration

cNIs were administered to dogs by the site’s veterinary ultrasonographer and PI as follows: an i.v. catheter was placed, and the dog was sedated with dexmedetomidine (5 mcg/kg) and butorphanol (0.1–0.2 mg/kg). Abdominal fur was shaved, and skin was prepared for aseptic injection. NIs were warmed to room temperature, and an 18 gauge, sterile cannula was placed through the linea alba, ~ 3–4 cm cranial to the umbilicus, under ultrasound guidance to intraperitoneally infuse normal saline test solution, then the suspended cNIs over a 2–3 minute period. Once the administrations were completed, the cannula was withdrawn. Ultrasound imaging was carried out to check for abdominal bleeding. Sedation was reversed with atipamazole (Antisedan), and the dog was monitored until determined stable by the PI and veterinary staff. Dogs returned home with their owners the same day once the PI determined that they were stable and ready. Owners were advised that the dogs’ serum glucose concentrations should be kept ≤ 210 mg/dL to protect the graft cells and facilitate redifferentiation into insulin producing cells.

### Planned follow-up schedule per full protocol (note, no dog is yet fully 3 years post-treatment)

#### Month 1

Dog owners check and record their dog’s blood glucose levels every 12 hours, and record food and water intakes and weights once per week on supplied forms. Dog owners administer insulin to dogs as needed and as instructed by the PI. Dogs are brought in for a physical examination and laboratory studies as indicated in S2 Table.

#### Months 2–12

Dog owners continue to check and record their dog’s blood glucose levels twice per day; record dogs' daily food and water intakes and weights every other week on supplied forms and electronically through month 6, and then as deemed necessary by the PI. Dog owners are responsible for the continued administration of insulin to dogs as needed and as instructed by the PI. Dogs are brought in for a physical examination once per month post treatment through month 6, and once per quarter in months 6–12. Fructosamine levels, a chemistry panel and urinalysis are obtained at the 3rd and 6th month visits. At the 6 months visit, a CBC, Serum chemistry and electrolyte panel, HbA1c, and urine are collected. At each visit, potential changes in the degree of preexisting end-organ damage are carefully documented.

#### Years 1–3

Dog owners are responsible for continued checking of blood glucose levels and administering insulin as prescribed by the PI. Dogs are brought in for a physical examination once per quarter through the 36th month post treatment, to assess changes in the degree of documented end-organ damage. Fructosamine levels, HbA1c and chemistry panels are checked at each visit. Once per year, a CBC and a urinalysis are obtained.

### Statistical analysis

Unless otherwise indicated, data are expressed as mean ± SEM or mean ± 95% confidence interval, as indicated. Primary data were collected using Excel (Microsoft, Redmond, WA), and statistical analyses were carried our using Prism (GraphPad, San Diego, CA). Two tailed t-tests were used to assess differences between data means. A *P* value of < 0.05 was considered significant. For rtPCR, data are presented as Log10RQ, and statistical significance is defined as ≥ ± 2, as described in [29].

## Results

### Study design

This FDA-CVM guided Pilot study to determine the safety, feasibility and preliminary efficacy of cNIs in eliminating or significantly reducing a diabetic dog's need for insulin is being carried out as detailed in Methods. Six dogs have been administered allogeneic cNIs. The dogs’ pretreatment demographics and comorbidities are summarized in Tables 2 and 3. Two dogs are being treated for hypertriglyceridemia (gemfibrozil, 150 mg BID, and dietary restriction) and have been treated but are not yet in the intermediate term follow-up phase of the study, while four have been treated with cNIs (VSH-01, VSH-02, WSU-01 and WSU-02) and have been followed for more than 6 months. VSH-01 was a 9 year old (at dosing), male, 12 kg French bulldog who had been on insulin for approximately 2.5 years at the time of dosing. VSH-02 was a 7 year old, hypothyroid, male, 7 kg Bichon mix who had been diabetic for approximately 6 months at the time of dosing. WSU-01 was a 2 year old, 11 kg, female American Eskimo dog who had been on insulin for approximately 9 months prior to treatment. WSU-02 was a 1 year old, 7 kg Chihuahua mix who had been on insulin for approximately 6 months prior to dosing.

**Fig 1:**
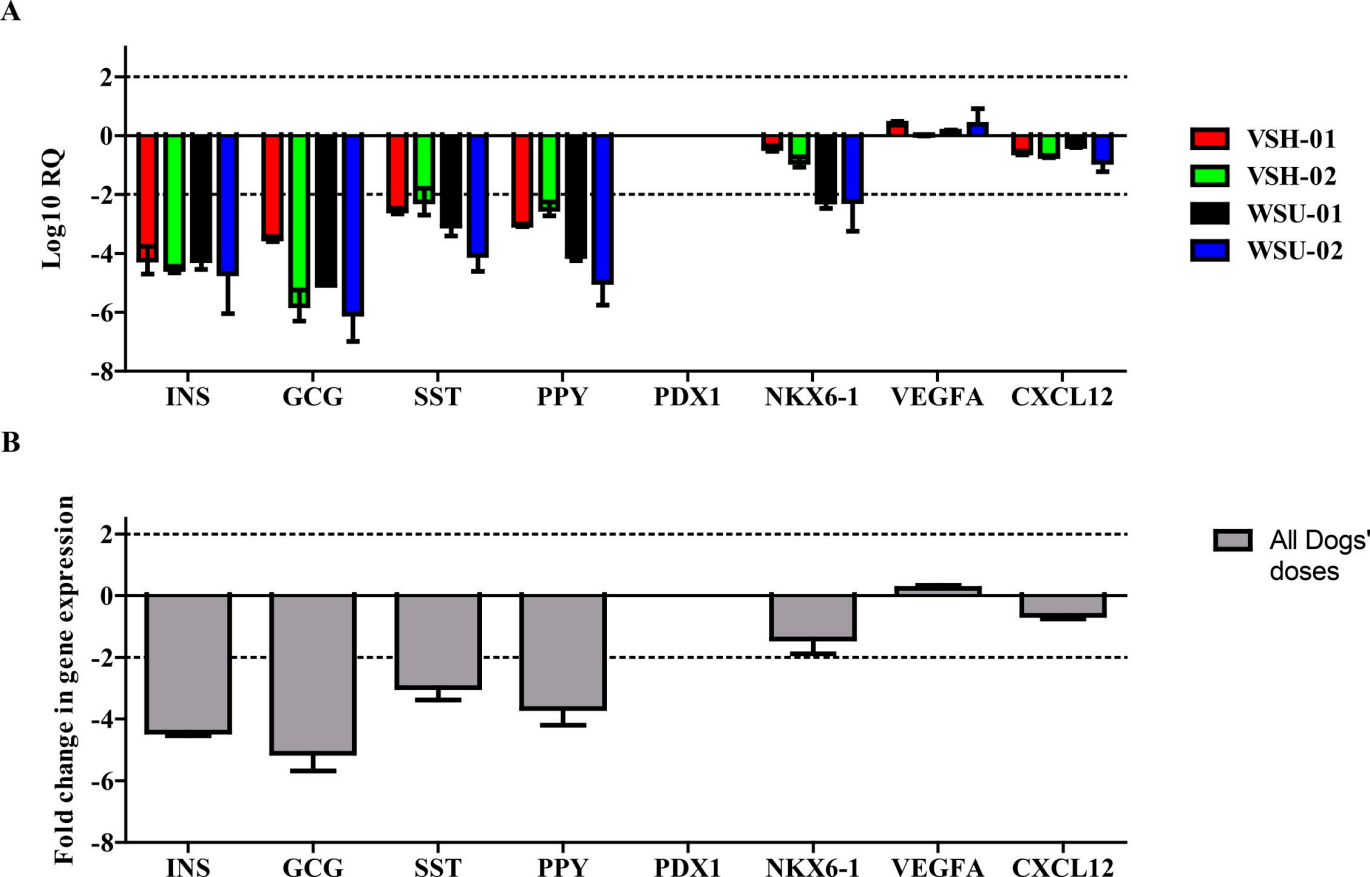
Gene expression profiles of the cNIs administered to study subjects. (A) All gene expression levels were normalized to those of the P0 IC banks from which the IC component of the cNIs were derived and are given here as Log10 Relative Quantitation (RQ; see Methods). Data are expressed as mean with 95% confidence interval, and all reactions were carried out in duplicate. INS, GCG, SST, PPY, NKX6-1, VEGF, and CXCL12 are all expressed in the cNIs administered to each dog. PDX-1 is no longer expressed. (B) Fold changes in gene expression (mean ± SEM) of the doses given to all dogs combined, versus those of the P0 IC banks from which the IC components were derived. For all treated dogs, their cNI doses express ~4-fold less insulin, ~5-fold less GCG, ~3-fold less SST, ~4-fold less PPY than the P0 ICs from which they were partially derived. Aside from PDX-1, which was not detected in cNI doses, other assessed expression profiles (NKX6-1, VEGF, and CXCL12) were not significantly different from those of the P0 ICs. For both (A) and (B), a difference of ± 2 is considered significant (see Methods).

Therapeutic doses of cNIs for all treated dogs were prepared, release-tested, packaged and administered as described in Methods. All cells and final products met the release criteria listed in Table 4. Prior to administration, cNIs were characterized by rtPCR for gene expression of INS, GCG, SST, PPY, PDX-1, NKX6-1, VEGFA, and CXCL12. As shown in Fig 1, while PDX-1 was no longer detectible in cNIs given to any dog, cNIs used for treatment transcribed islet hormone genes for INS, GCG, SST and PPY albeit at significantly reduced levels compared to P0 cultured islet cells. cNIs also expressed genes associated with ASCs cytoprotective, angiogenic and immune modulatory activities [31–36] (Fig 1).

### Intermediate term blood glucose levels and insulin requirements

**Fig 2:**
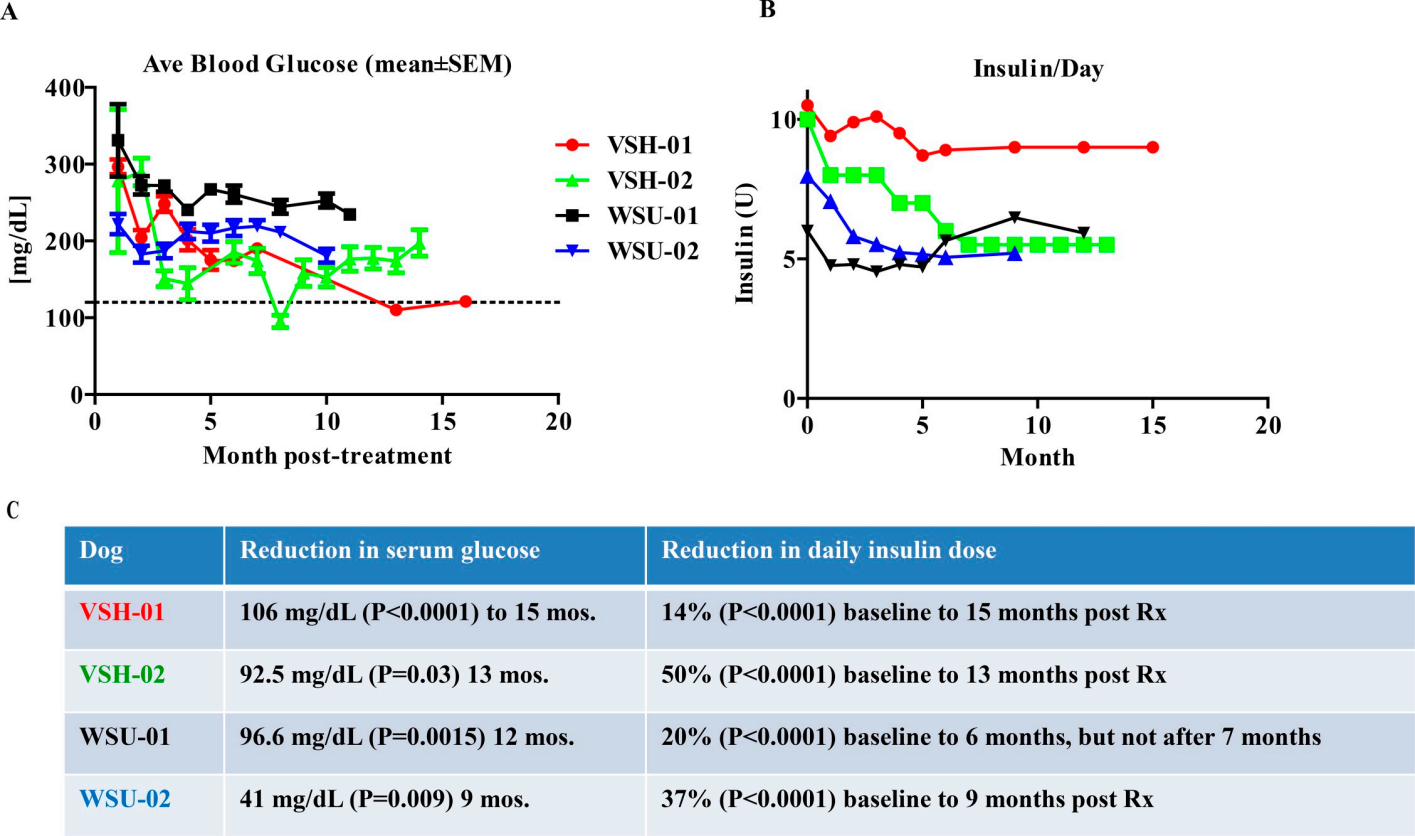
Serum glucose levels and insulin needs over time. (A) Serum glucose concentration of study dogs, as assessed and reported by owners, prior to treatment (0 months) and over the study period. As dogs were treated at different times, they are not all currently at the same post-treatment time, thus the reported follow-up period for each is different. (B) Insulin doses for the same time frame. Glucose and insulin dose are reported at leasttwice per day. All values for glucose concentration are averaged for the pretreatment period and for each month post-treatment. Units of insulin administered per day are calculated and averaged for each month. (C) Percent reduction in daily insulin dose at the current follow-up point from baseline and average mg/dL reduction in serum glucose from baseline along with statistical significance (P values) for each dog are shown.

As shown in Fig 2, blood glucose concentrations and insulin requirements for VSH-01, VSH-02, and WSU-02 were significantly reduced (P < 0.05) ≥ 6 months post treatment compared to baseline. Although WSU-01’s mean monthly blood glucose concentrations 12 months post treatment were also significantly reduced compared to those at baseline (P = 0.0015), her daily insulin needs remained unchanged (Fig 2). With the exception of WSU-01, all treated dogs currently show both a sustained reduction in serum glucose concentrations and insulin doses. WSU-01 showed continued reduction in serum glucose concentrations for her entire 12 month follow-up period, but only demonstrated a 20% reduction in insulin dose over 6 months post-treatment. After 7 months, her needed insulin dose rebounded to her pretreatment levels and remains there now (see Fig 2).

### Markers of intermediate-term glucose control

With the exception of VSH-02, all dogs’ fructosamine levels were in the 200–400 μmol/L range both prior to and post-treatment, indicating consistent and good control of glucose levels on the parts of all owners. For VSH-02, pretreatment fructosamine was 439 μmol/L, and deceased post treatment to 285 μmol/L, which is within normal range. VSH-01’s fructosamine levels also decreased post-treatment to 297 μmol/L (within normal range). While HbA1c levels were called for in the protocol, they have been inconsistently recorded. However, VSH-02’s HbA1c decreased from 12.1 pre-treatment to 6.8% post-treatment.

### Type of diabetes

**Fig 3:**
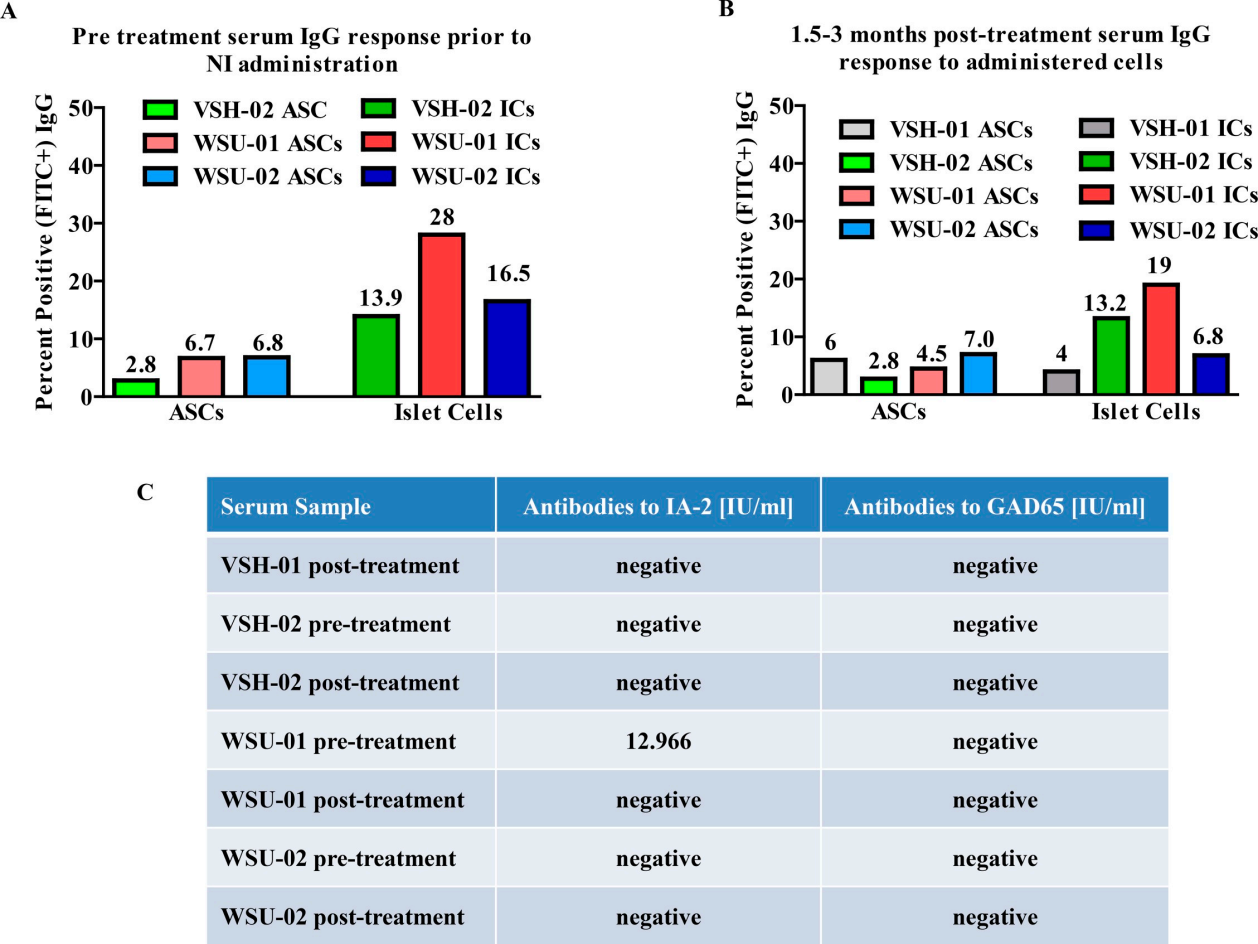
Antigenic responses to cNI treatment, and pre-existing presence of auto-islet antibodies. AntiASC and anti-IC IgG responses as assessed by FACS in sera of the study dogs before (A) and after (B) treatment with allogeneic cNIs. Shown are percentages of FITC-labeled anti-dog IgG antibody. Sera were collected from dogs before (A) and 1.5 to 3 months after (B) cNI administration. The percent of positive cells is indicated above each column. Dogs do not show increased IgG responses to either ASCs or ICs after cNI administration, indicating there is no additional allo-immune response by the recipients to either cell type. Three dogs show pre-existing antibodies to ICs prior to treatment with cNIs, suggesting they have an autoimmune form of diabetes. (C) Results of ELISA testing of sera from the treated dogs for specific anti-islet antigens, IA2 and GAD65, indicate that none of the dogs have antibodies to GAD65 antigen, but that WSU-01’s serum contained antibodies to IA2 prior to, but not after treatment. Samples were run in duplicate. Note: a pre-treatment serum sample was not available for VSH-01.

As part of the study, serum from dogs was obtained prior to and after cNI dosing, and tested by FACS for antibodies to the administered cells, using pre-dosing serum and isotypes as negative controls. Pre-treatment testing of sera served not only the purpose of giving a baseline level of anti-ASC or anti-IC antibodies to compare to post-treatment levels, but also as an indication of whether the dogs’ diabetes was auto-immune in nature, as indicated by the presence of anti-IC antibodies prior to treatment. By this criterion, three of the four tested dogs, VSH-02, WSU-01 and WSU-02, appear to have autoimmune diabetes (see Fig 3A and 3B), while VSH-01 may have an “insulin resistant” form of insulin-dependent DM.

Sera were similarly tested for the presence of antibodies to two common human autoimmune antigens that have been associated with canine T1DM, IA2 and GAD 65 [16,17]. Only pretreatment serum from WSU-01 showed reactivity to IA2. No other sera showed the presence of antibodies to either (Fig 3C).

### Neither auto- nor allo-immune rejection is observed

Even though at least 3 of the treated dogs (VSH-02, WSU-01 and WSU-02) appear to have autoimmune diabetes (Type 1) as indicated by the presence of auto-anti-islet cell IgG in their sera (see Fig 3), and even though the cNIs used to treat them came from unrelated donors, none of the dogs appear to have rejected the cNIs as indicated by the following. First, responsive dogs show continued improved blood glucose concentrations and lowered insulin requirements (see Fig 2). Second, no allo-rejection antibodies to the cNIs are found in the treated dogs’ sera after implantation (Fig 3).

### cNI therapy appears to be safe and well tolerated in dogs

In addition to regular blood glucose and weight monitoring, dogs enrolled in this study are being, and will continue to be, followed closely over the entire protocol-designated 3 year follow-up period with physical examinations and laboratory tests in order to detect signs of adverse events or changes in end organ damage in association with Neo-Islet therapy (see S2 Table).

Despite the fact that several of the currently studied dogs are of advanced age and have multiple comorbidities (see Tables 2 and 3), no adverse events attributable to the cNI therapy have been observed to date. Specifically, none of the 6 treated dogs have developed adverse events such as oncogenic transformation of transplanted cells, hematological changes, deterioration in organ function, failure to thrive, etc.. While at this point in the study we cannot rule out with certainty the possibility that adverse events can eventually occur, data in the nearly 2 years since the first dogs were treated would indicate that cNIs are safe and well tolerated.

## Discussion

Intermediate term results from the current study thus far demonstrate that allogeneic cNI therapy, as currently dosed, (i) is effective in improving glycemic control while durably reducing insulin doses; (ii) it does so without eliciting an immune response, even in dogs with autoimmune diabetes; and (iii) is feasible and safe. The observed decrease in post-treatment compared to pre-treatment levels of anti-IC antibodies in two dogs (Fig 3A) may reflect the known inhibitory actions of ASCs on B cells [37]. However, this potentially significant effect must be confirmed in additional studies. While the documented reduction in total insulin requirement occurs only gradually as transplanted ICs, we postulate, re-differentiate, as was demonstrated in our previous publication [21] and in S1 Fig, into insulin producing cells, this response does taper off subsequently. However and importantly, the insulin dose does not, in most cases, increase again (Fig 2) as is frequently seen in failing, traditional intrahepatic islet cell transplants [38]. Significantly, we previously demonstrated in cNI treated, euglycemic, STZ-diabetic NOD/SCID mice, intraperitoneal glucose tolerance tests were of normal pattern compared to controls, and exclusively canine insulin was released [21]. Further, we show here (see S1 Fig) that cNI retrieved from euglycemic, STZ-diabetic, cNI treated NOD-SCID mice 9 weeks post-treatment secrete significantly increased quantities of insulin in response to glucose. These data clearly demonstrated that in this xenogeneic diabetic mouse model, cNIs redifferentiate in vivo into insulin producing cells [21]. In the present study, we cannot rule out the possibility that adherence to the protocol itself may contribute to the dogs’ improved glycemic control. However, the fact that this is accompanied by a decrease in the overall need for exogenous insulin long term argues that the cNIs are contributing needed insulin. Although we have not conducted studies on the engrafted cNIs in these pet dogs, we postulate that the mechanism of action here is the same as was seen in the mice: that the cNIs redifferentiate in vivo to provide insulin in these diabetic dogs.

The data so far indicate that the allogeneic cNI grafts are stable and functioning long term, and are not being rejected, which directly demonstrates that this novel form of therapy does not require the life-long use of potentially toxic antirejection drugs. In other words, the allogeneic ASC component of the administered cNIs appears to provide through its immune-modulating activities [37] both robust auto- and allo-immune isolation of the cells that make up cNIs, and this without the need for often fail-prone encapsulation devices. For example, the use of such a subcutaneously implanted encapsulation device in a clinical trial has proved problematic, as it elicited an inflammatory fibrotic, foreign body type response that resulted in the death of the encapsulated insulin producing cells and thus failure so far of this mode of T1DM therapy [39].

Since the treated dogs are pets and the study is ongoing, the exact engraftment site of the i.p. administered cNIs has not been histologically confirmed, although we believe that the main engraftment site is the omentum as we clearly demonstrated in our mouse studies that also used cNIs (21). Late post treatment glucose tolerance tests with simultaneous monitoring of canine insulin and C-Peptide release will be conducted in all study dogs.

There are several possible explanations for the incomplete normalization of blood glucose concentrations and failure to achieve complete insulin independence, contrary to what was found in mice treated with cNIs [21]. Incomplete responses may be related to an inadequate cNI dose, the potential need for a second dose, as is routinely done in human islet transplants [38] and as we demonstrated to be effective in incompletely controlled diabetic mice (unpublished observations), and potentially suboptimal omental uptake and engraftment of cNIs. In addition, the need to keep the dog post cNI infusion for at least 24 hrs either in a prone or supine position may be important since both of these positions facilitate the omental engraftment of administered cNIs, while the assumption of an upright position will lead to the translocation of the transplants to the dog’s pelvis, a location that prevents their engraftment in the omentum and failure to function as intended [40]. The current technology exploits the omentum’s ability to both release cells and to take up cell aggregates such as cNIs via its milky spots, combined with its excellent arterial blood supply for oxygenation of and glucose sensing by engrafted cNIs. Importantly, the omentum’s venous drainage facilitates the physiological delivery of secreted insulin and other islet hormones directly into the portal system of the liver, i.e., a route that is identical to that of the pancreatic veins. Since the liver inactivates up to 50% of received insulin, the post-hepatic concentrations of insulin that other insulin-sensitive tissues are exposed to is significantly lower than those insulin levels that are generated by the s.c. injection of insulin that, particularly in higher doses, may have adverse systemic effects [41–43].

Finally, the ability to generate potentially more than 80 therapeutic cNI doses from a single cadaveric pancreas and ASC donor will significantly improve the availability of this therapy for diabetic dogs and thus assist their owners with the care of their pets. Once the therapy has been fully optimized to durably make diabetic dogs insulin independent, the cost savings over timeare predicted to be significant.

In conclusion, completion of the current study with the remaining dogs will include permitted protocol modifications that we expect to augment the therapeutic efficacy of the so far utilized cNI treatment protocol. Nevertheless, we posit that the observations presented herein provide further evidence in support of our hypothesis and previously published work that NIs when given i.p. engraft in the omentum where they redifferentiate and create a new endocrine pancreas that leads to the establishment of euglycemia and insulin-independence [21]. The proof of principle, i.e., the demonstration that this NI therapy is also effective in a second, larger diabetic mammal, the dog, is definitively significant as this will further strengthen the justification for the currently planned conduct of a scientifically based clinical trial in human study subjects with T1DM.

## Supporting information

S1 Table PCR reagents used and their sources. (DOCX)

S2 Table Follow-up testing schedule. (DOCX)

S1 Fig Glucose Stimulated Insulin Secretion (GSIS) of cNIs before and after implantation in mice. GSIS per cNI of freshly formed cNIs vs. cNIs retrieved from euglycemic, STZ-diabetic, cNI-treated NOD-SCID mice 9 weeks post cNI administration. *, *P* < 0.05 compared to 5 mM glucose; **, P < 0.01 compared to pre-administration. These animals data were from those reported in [21].
(TIF)

S1 File Methods for GSIS in freshly prepared cNIs vs. retrieved cNIs. (DOCX)
