# Supplementary material for: Interim report on the effective intraperitoneal therapy of insulin-dependent diabetes mellitus in pet dogs using “Neo-Islets,” aggregates of adipose stem and pancreatic islet cells (INAD 012-776)
Source: PLoS One. 2019 Sep 19;14(9):e0218688. doi: 10.1371/journal.pone.0218688 (PMC6752848; doi:10.1371/journal.pone.0218688)
Supplement: S1 Table — (DOCX) [file pone.0218688.s001.docx]

**S1 Table.** PCR Reagents used and their sources.

| **Reagent** | **Supplier** | **Catalog #** |
| --- | --- | --- |
| Rneasy Mini Kit | Quiagen | 74107 |
| SuperScript II reverse transcriptase | ThermoFisher Scientific | 18064014 |
| Random Primers | ThermoFisher Scientific | 48190011 |
| 100mM dNTP Set | ThermoFisher Scientific | 10297018 |
| TaqMan Universal Master Mix II with UNG | ThermoFisher Scientific | 4440038 |
| Water Nuclease-free | Invitrogen | AM9937 |
| PCR Plate | Life Technologies | 4306737 |
| Adhesive film | ThermoFisher Scientific | 4311971 |
|  |  |  |
| **PCR Primer for Gene** | **Vendor** | **Catalog #** |
| Beta Actin (ACTB) | Life Technologies | Cf03023880_g1 |
| Beta 2 Microglobulin (B2M) | Life Technologies | Cf02659077_m1 |
| Stromal Cell Derived Factor 1 (CXCL12) | Life Technologies | Cf02625258_m1 |
| Vascular Endothelial Growth Factor A (VEGFA) | Life Technologies | Cf02623449_m1 |
| Insulin (INS) | Life Technologies | Cf02647520_m1 |
| Glucagon (GCG) | Life Technologies | Cf02624195_m1 |
| Pancreatic and Duodenal Homeobox 1 (PDX1) | Life Technologies | Cf02622671_m1 |
| NK6 Homeobox 1 (NKX)6-1 | Life Technologies | Cf02705682_mH |
| Somatostatin (SST) | Life Technologies | Cf02625293_m1 |
| Pancreatic Polypeptide (PPY) | Life Technologies | Cf02653446_g1 |
