# Supplementary material for: Interim report on the effective intraperitoneal therapy of insulin-dependent diabetes mellitus in pet dogs using “Neo-Islets,” aggregates of adipose stem and pancreatic islet cells (INAD 012-776)
Source: PLoS One. 2019 Sep 19;14(9):e0218688. doi: 10.1371/journal.pone.0218688 (PMC6752848; doi:10.1371/journal.pone.0218688)
Supplement: S2 Table — (DOCX) [file pone.0218688.s002.docx]

**S2 Table. Follow-up testing schedule.**

| Test/Visit |  |  | Month post treatment | | | | | | | | | | | | | | | |
| --- | --- | --- | --- | --- | --- | --- | --- | --- | --- | --- | --- | --- | --- | --- | --- | --- | --- | --- |
|  | Pretreatment | treatment | 1 | 2 | 3 | 4 | 5 | 6 | 9 | 12 | 15 | 18 | 21 | 24 | 27 | 30 | 33 | 36 |
| UA with protein dipstick | x |  |  |  |  |  |  | x |  | x |  |  |  | x |  |  |  | x |
| UPC (if dipstick warrants)* | x |  |  |  |  |  |  |  |  |  |  |  |  |  |  |  |  |  |
| Urine Culture* | x |  |  |  |  |  |  |  |  |  |  |  |  |  |  |  |  |  |
| CBC with diff | x |  | x |  |  |  |  | x |  | x |  |  |  | x |  |  |  | x |
| Chemistry panel** | x |  | x |  | x |  |  | x | x | x | x | x | x | x | x | x | x | x |
| IgG Serum test | x |  | x |  |  |  |  | x |  |  |  |  |  |  |  |  |  |  |
| Chest xray (dogs >/= 9 years old)* | x |  |  |  |  |  |  |  |  |  |  |  |  |  |  |  |  |  |
| Fructosamine | x |  | x |  | x |  |  | x | x | x | x | x | x | x | x | x | x | x |
| HbA1c | x |  |  |  |  |  |  | x | x |  |  |  |  | x |  |  |  | x |
| Physical Exam | x | x | x | x | x | x | x | x | x | x | x | x | x | x | x | x | x | x |
| Blood glucose |  | x |  |  |  |  |  |  |  |  |  |  |  |  |  |  |  |  |
| Abdominal ultrasound* | x | x |  |  |  |  |  |  |  |  |  |  |  |  |  |  |  |  |
| Thyroid panel, if PI believes warranted* | x |  |  |  |  |  |  |  |  |  |  |  |  |  |  |  |  |  |
| cPLI, if PI believes warranted* | x |  |  |  |  |  |  |  |  |  |  |  |  |  |  |  |  |  |
| any other laboratory testing deemed pertinent by enrolling veterinarian* | x |  |  |  |  |  |  |  |  |  |  |  |  |  |  |  |  |  |
| *, as needed for duration of study |  |  |  |  |  |  |  |  |  |  |  |  |  |  |  |  |  |  |
| **, including: electrolytes (Na, K, Cl, Bicarbonate, Ca^++^, PO_4_), liver function tests, serum Creatinine, Blood Urea Nitrogen. | | | | | | | | | | | | | | | | |  |  |
