# Supplementary figures and images for: Interim report on the effective intraperitoneal therapy of insulin-dependent diabetes mellitus in pet dogs using “Neo-Islets,” aggregates of adipose stem and pancreatic islet cells (INAD 012-776)
Source: PLoS One. 2019 Sep 19;14(9):e0218688. doi: 10.1371/journal.pone.0218688 (PMC6752848; doi:10.1371/journal.pone.0218688)

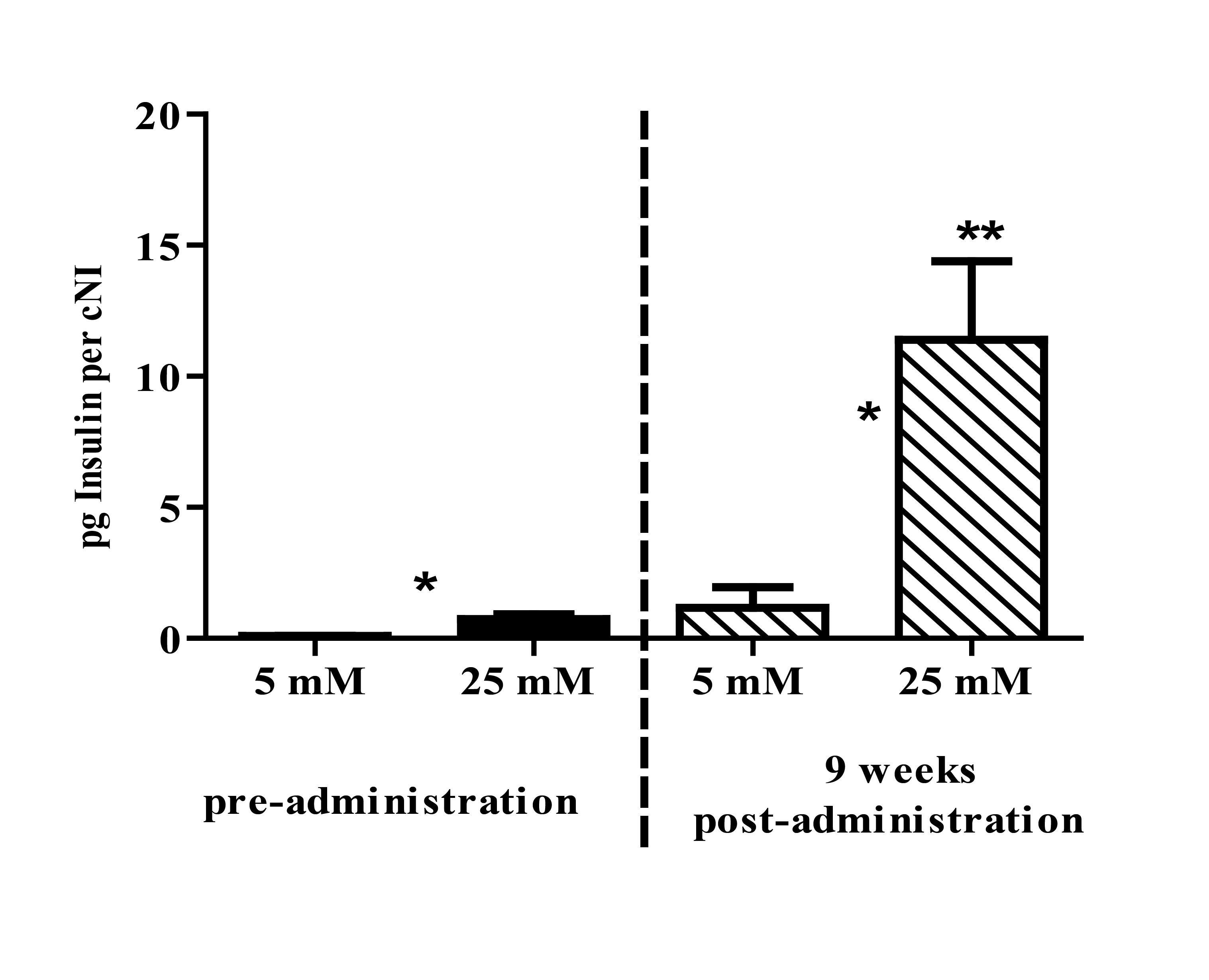

Supplement: S1 Fig — GSIS per cNI of freshly formed cNIs vs. cNIs retrieved from euglycemic, STZ-diabetic, cNI-treated NOD-SCID mice 9 weeks post cNI administration. *, P < 0.05 compared to 5 mM glucose; **, P < 0.01 compared to pre-administration. These animals data were from those reported in [21]. (TIF) [file pone.0218688.s003.tif]
