# Supplementary material for: Interim report on the effective intraperitoneal therapy of insulin-dependent diabetes mellitus in pet dogs using “Neo-Islets,” aggregates of adipose stem and pancreatic islet cells (INAD 012-776)
Source: PLoS One. 2019 Sep 19;14(9):e0218688. doi: 10.1371/journal.pone.0218688 (PMC6752848; doi:10.1371/journal.pone.0218688)
Supplement: S1 File — (DOCX) [file pone.0218688.s004.docx]

**Methods for GSIS in freshly prepared cNIs vs. retrieved cNIs.**

cNIs were tested for their ability to secrete insulin in response to glucose (GSIS) as compared to retrieved islets (see S1 Fig). GSIS was conducted as follows: (a) 1000 freshly formed cNIs were collected or (b) retrieved NIs were collected from euglycemic, cNI-treated, STZ diabetic NOD-SCID mice 9 weeks post cNI administration (n=2), suspended in 1 ml DMEM 5 mM glucose (Gibco 11885-084), and incubated at 37°C for 1 hour, after which the NIs were collected and the supernatant discarded. The cNIs were resuspended in 1 ml DMEM 5mM glucose, and incubated again at 37°C for 1 hour, after which the supernatant was collected and stored at -20°C until ready for analysis. The high and low glucose supernatants were assessed for insulin content using an ELISA kit and following the manufacturer’s instructions (Crystal Chem 90050). Both freshly formed and retrieved cNIs were found to secrete insulin in response to glucose stimulation. Retrieved cNIs secrete 15-fold higher concentrations of insulin than do freshly formed cNIs. Freshly formed cNIs, being made from culture expanded ICs, are expected to secrete lower levels of insulin as they are partially dedifferentiated [1,2]. cNI-treated, STZ-diabetic, euglycemic NOD-SCID mice were those detailed and described in our previous publication [3]. See that reference for animal study methods.

**References**

1. Joglekar M V, Hardikar A. Epithelial-to-mesenchymal transition in pancreatic islet β cells. Cell Cycle. 2010;9: 4077–4079. doi:10.4161/cc.9.20.13590

2. Russ H a, Ravassard P, Kerr-Conte J, Pattou F, Efrat S. Epithelial-Mesenchymal Transition in Cells Expanded In Vitro from Lineage-Traced Adult Human Pancreatic Beta Cells. Maedler K, editor. PLoS One. 2009;4: e6417. doi:10.1371/journal.pone.0006417

3. Westenfelder C, Gooch A, Hu Z, Ahlstrom J, Zhang P. Durable Control of Autoimmune Diabetes in Mice Achieved by Intraperitoneal Transplantation of “Neo-Islets,” Three-Dimensional Aggregates of Allogeneic Islet and “Mesenchymal Stem Cells”. Stem Cells Transl Med. 2017;6: 1631–1643. doi:10.1002/sctm.17-0005
